# Diabetes and Hepatitis C: A Two-Way Association

**DOI:** 10.3389/fendo.2015.00134

**Published:** 2015-09-14

**Authors:** Sara Salehi Hammerstad, Shira Frankel Grock, Hanna J. Lee, Alia Hasham, Nina Sundaram, Yaron Tomer

**Affiliations:** ^1^Department of Medicine, Division of Endocrinology, Icahn School of Medicine at Mount Sinai, New York, NY, USA; ^2^Department of Pediatrics, Oslo University Hospital Ullevål, Oslo, Norway; ^3^James J. Peters VA Medical Center, Bronx, NY, USA

**Keywords:** type 1 diabetes, type 2 diabetes, hepatitis C virus, hepatitis C, insulin resistance, interferon alpha, hepatocellular carcinoma, outcomes, treatment

## Abstract

Diabetes and hepatitis C infection are both prevalent diseases worldwide, and are associated with increased morbidity and mortality. Most studies, but not all, have shown that patients with chronic hepatitis C are more prone to develop type 2 diabetes (T2D) compared to healthy controls, as well as when compared to patients with other liver diseases, including hepatitis B. Furthermore, epidemiological studies have revealed that patients with T2D may also be at higher risk for worse outcomes of their hepatitis C infection, including reduced rate of sustained virological response, progression to fibrosis and cirrhosis, and higher risk for development of hepatocellular carcinoma. Moreover, hepatitis C infection and mainly its treatment, interferon α, can trigger the development of type 1 diabetes. In this review, we discuss the existing data on this two-way association between diabetes and hepatitis C infection with emphasis on possible mechanisms. It remains to be determined whether the new curative therapies for chronic hepatitis C will improve outcomes in diabetic hepatitis C patients, and conversely whether treatment with Metformin will reduce complications from hepatitis C virus infection. We propose an algorithm for diabetes screening and follow-up in hepatitis C patients.

## Introduction

Type 2 diabetes (T2D) and hepatitis C are prevalent diseases worldwide. The World Health Organization (WHO) reported that 170 million people are chronically infected with hepatitis C virus (HCV) globally (1), and 347 million have diabetes mellitus (DM). Despite decreasing prevalence of hepatitis C infection in the United States, the disease burden continues to grow due to hepatitis C-related diseases (2). Studies have shown that more than one-third of patients with chronic HCV infection will develop at least one extrahepatic manifestation (3, 4).

A large number of studies report an increased risk for T2D in patients with chronic HCV infection (5). While a few studies could not confirm this association in the absence of liver dysfunction (6), the majority of studies strongly support this association. This association holds even when comparing patients with chronic HCV to patients with other progressive viral liver diseases (7, 8). There are also studies reporting T2D as a predisposing factor for HCV infection (9–11).

While insulin resistance (IR) and T2D are more frequently reported as complications of HCV infection, HCV is also known to be associated with several autoimmune manifestations, including type 1 diabetes (T1D) (12, 13). Therapy for chronic HCV infection, in particular interferon alpha (IFNα), can also trigger diabetes. The immunomodulatory effect of IFNα can induce or exacerbate autoimmune diseases. While interferon-induced thyroiditis (IIT) is the most frequently reported autoimmune complication of IFNα therapy (14, 15), other autoimmune conditions, such as T1D (16), systemic lupus erythematosus (17), rheumatoid arthritis (18), pernicious anemia (19), optic neuritis (20), vitiligo (21), and autoimmune hemolytic anemia (22) are also associated with HCV infection [for review, see Ref. (23)].

Recently, new non-interferon-based therapeutic regimens for hepatitis C have been introduced; however, chronic HCV infection and its sequelae remain a serious public health concern especially cirrhosis and hepatocellular carcinoma (HCC) which are the leading causes of death in patients with chronic HCV (24). Numerous studies illustrate that IR and T2D have a negative impact on clinical outcomes for patients with chronic HCV infection and it is possible that insulin-sensitizing therapy with metformin might be beneficial for reducing HCV complications. In this review, we analyze the literature linking HCV infection and its therapy – IFNα – with diabetes. We focus on the mechanisms by which both HCV infection and IFNα treatment may contribute to the development of T2D and T1D (in this review, we refer to all forms of autoimmune diabetes as T1D), and the mechanisms by which diabetes can worsen outcomes in HCV infection.

We systematically searched PubMed databases from 1990 to 2015.The search was limited to studies reported in English. The reference lists from identified studies were further searched for primary sources.

## Type 2 Diabetes and Insulin Resistance in HCV Infection

### Hepatitis C infection, insulin resistance, and type 2 diabetes

An association between cirrhosis and glucose intolerance has long been known. Indeed, cirrhosis itself is “diabetogenic” with various studies describing the majority of cirrhotics as having impaired glucose tolerance (25–27). Beginning with Allison’s group in 1994 (26), epidemiological studies have since specifically linked HCV and HCV cirrhosis with DM (25, 26). The prevalence of DM, the majority assumed to be T2D, was significantly higher among patients with HCV cirrhosis than in patients with cirrhosis due to other etiologies (26). HCV infection precedes the diagnosis of T2D in as many as 73% of cases, further suggesting HCV’s pathogenic role in the development of T2D (28–30).

It is estimated that up to 33% of chronic hepatitis C patients have T2D (31). One meta-analysis of 34 studies showed a significantly higher risk for T2D in HCV patients are compared to hepatitis B virus (HBV) patients [odds ratio (OR) 1.8], matched controls, and patients with other forms of chronic liver disease infection (5). Perhaps due to subtle variations in HCV genotype, ethnicity, severity of liver disease, and other variables, a few studies have found no association (32–34). Mangia et al. prospectively followed 385 non-cirrhotic hospitalized patients and concluded that the prevalence of DM was not significantly different than that in the general Italian population. A recent NHANES study also had conflicting results and showed that HCV infection was only associated with ALT and GGT elevations, not diabetes. However, there was still a clear trend for higher homeostatic model assessment-estimated insulin resistance (HOMA-IR) scores in both the HCV antibody and RNA-positive groups compared to controls (35). Regardless, the vast majority of studies have noted a 2- to 10-fold increase of T2D in chronic HCV infection compared to other liver diseases (31, 36–38). The cumulative data suggest that T2D is approximately two to threefold more prevalent in HCV than in HBV infection. We therefore believe that the risk for T2D among HCV patients is significant and is more than just coincidental or cirrhosis-dependent (5, 39, 40).

#### Epidemiology

A positive association between chronic HCV infection and T2D has been consistently demonstrated across different ethnicities and geographic regions, in both developed and developing countries. The overall prevalence of DM among chronic HCV-seropositive populations in North America, Europe, the Middle East, and Asia ranges from 13 to 33% (39, 41, 42). It is estimated that 20% of chronic HCV patients will develop cirrhosis and as many as ~50% of these patients will have T2D (26, 40). This translates into 47 million people worldwide and 750,000 people in the USA alone who are expected to develop HCV-associated T2D (31).

The landmark cross-sectional NHANES III study that surveyed 9,841 US adults concluded that HCV-positive U.S. patients older than 40 years old had a threefold increased risk for T2D, compared to those without HCV (OR 3.77) (43). Other studies from the U.S. and abroad have corroborated this association between hepatitis C and T2D (7). The highest rate of HCV infection in the world is found in Egypt where 12% of its general population and up to 40% of its older, rural population is HCV seropositive (41). The prevalence of DM is 25.4% among the HCV population in Egypt which indicates that chronic HCV patients are three times more likely to develop DM than the HCV seronegative population (38). In Italy, the frequency of HCV infection varies depending on certain geographic regions but ranges from 3 to 15% in the general population (44). One retrospective study showed that the relative risk for T2D in non-cirrhotic HCV patients was as high as 2.71 compared to that of non-cirrhotic HBV patients and 1.81 compared to controls without hepatitis (45). Likewise, the prevalence of DM (presuming that the majority are type 2 DM) in South Korea is significantly increased in the chronic HCV patients (23.5%) compared to chronic HBV patients (8.2%) (46). In a Japanese retrospective study, the prevalence of DM (also presumed to be mostly T2D) was 20.9% among the non-cirrhotic HCV patients versus 11.9% among the non-cirrhotic HBV patients (47). Among the cirrhotic population, the prevalence rates for diabetes showed analogous findings; DM was found in 30.8% of the HCV cirrhotics versus 11.9% of the HBV cirrhotics (47). The general trend, regardless of country of origin, suggests that T2D is approximately two to threefold more prevalent in HCV than in HBV infection.

The association between hepatitis C and T2D is also seen in the reverse direction, namely that diabetics are more prone to acquiring HCV. Although two small cohorts from Turkey and Nigeria failed to demonstrate an increased prevalence of hepatitis C among T2D patients, the vast majority of studies do. For instance, one US study showed the prevalence of hepatitis C to be 4.2% among diabetics compared to only 1.2% among the non-diabetic controls (7). By contrast, the prevalence of HBV in diabetics (0.3%) was not significantly increased compared to non-diabetics. A study in Taiwan that included 820 type 2 diabetics showed that HCV seropositivity was 2.8 times greater among T2D patients than in non-diabetic controls (48). Similarly, a study from Italy showed that the prevalence of HCV infection among 1,516 T2D patients was 7.6% compared to 2.3% in the non-diabetic controls (49). Comparable trends were also observed in Pakistan with an OR for HCV infection of 3.03 among T2D patients (50).

#### Risk Factors for Type 2 Diabetes in HCV Patients

##### Age and gender

Age has been shown to be a risk factor and an independent predictor for the development of diabetes in chronic HCV patients, although not consistently in every study. When separated into quintiles by age, one study noted an increased frequency of diabetes in all HCV patients except in the youngest quintile that included those less than 38 years old (7). In the NHANES III cohort of almost 10,000 U.S. adults, the likelihood for T2D among HCV patients older than 40 years old was twofold compared to those who were younger than 40 years old (43). When further evaluated for confounders, including ethnicity, sex, body mass index (BMI), and socioeconomic factors, the OR for T2D increased to 3.77 (43). Moreover, in Egypt it was also noted that the diabetic HCV patients were older than the non-diabetics (mean age 48.1 versus 40.7 years old) (41). Longer duration of hepatitis C infection has also been noted to increase the risk for diabetes.

Hepatitis C is more common among men than women (51), and male gender is also associated with more hepatitis C disease progression to fibrosis and cirrhosis. It is difficult, however, to precisely tease out the relative contribution of gender itself from confounders like fibrosis and hepatosteatosis, which are themselves markers of more severe liver disease and are associated with higher BMI, larger waist circumference, and a higher HOMA index (34).

##### Family history

Family history of diabetes has been demonstrated to be a risk factor for the development of T2D in HCV patients as in the general population (52). One case-control study showed that, among 45 non-cirrhotic HCV-seropositive patients, 67% of those with T2D had a family history for diabetes versus only 7% of those without T2D (31). Another study showed a positive family history for diabetes in 29% of the HCV diabetic group while only 1.8% had pertinent family history in the non-diabetic group (40). This risk is comparable to the two to sixfold increased risk for T2D in the general non-HCV population who have a positive family history for diabetes (52). Across various ethnic groups (Pima Indians and populations in the US, Norway, South Africa, and the UK), the risk of developing diabetes is consistently increased among individuals with a positive family history and is up to 4.4-fold higher for individuals with a single diabetic parent (52).

##### Genotype

T2D in chronic HCV infection has also been correlated with specific HCV genotypes. In North America, ~70% of HCV patients are infected with genotype 1a and 1b while a mere 4% are infected with genotypes 2a (53). Despite these numbers, genotype 1 is relatively less associated with diabetes in the HCV population. For instance, one US case-control analysis showed that HCV genotype 2 was present in as many as 29% of 594 HCV-positive diabetic patients versus only 3% of the local HCV control patients (7). In Japan, HCV genotype 2 patients also have a higher rate of DM than genotype 1 (47). Non-genotype 3 HCV (particularly genotypes 1, 2, and 4) has been more closely associated with the development of T2D among the hepatitis C population, and interestingly, genotype 2a in particular has been preferentially associated with extrahepatic manifestations of HCV. Fewer cases of IR were consistently demonstrated among HCV genotype 3 patients, even after adjusting for confounders like BMI and stage of fibrosis (52, 54). However, this has not been consistently demonstrated in all studies (40). These discrepancies may be due to the differences in the number of patients with available genotype data.

##### Cirrhosis

Chronic HCV infection in the absence of cirrhosis or antiviral therapy is associated with T2D (7, 31, 40, 55). This indicates that it is not the stage of liver disease alone that influences the development of T2D. The presence of fibrosis and cirrhosis, however, in some studies has been shown to be an independent risk factor that contributes to the progression to T2D (31, 33, 40, 42). Various studies have shown that the rate of T2D is higher among HCV cirrhotics than in other forms of cirrhosis, including cirrhosis caused by HBV, alcohol, or cholestatic disease (5, 40). Seventy to eighty percent of HCV cirrhotics have been reported to have glucose intolerance (31), and 50% of these cirrhotics have developed DM compared to only 9% of the non-HCV cirrhotics (26). This suggests a direct diabetogenic effect of the HCV beyond the damage to the liver. Furthermore, the rates of DM are even more increased on the background of higher grades of hepatic fibrosis, steatosis, or cirrhosis (31, 33, 40, 42, 56). For example, the prevalence of T2D was shown to increase with every rise in the fibrosis score of HCV patients with an OR of 3.83 (51). One prospective case series with 361 HCV patients from Pakistan showed an OR for T2D of 2.0 in HCV cirrhotics compared to that of HCV non-cirrhotics. Conversely, DM itself has recently been demonstrated to be hasten the progression of fibrosis and cirrhosis underscoring the bidirectionality of the association between chronic HCV infection and IR in chronic HCV infection (57) (see Hepatic Fibrosis and Cirrhosis).

##### Therapeutic regimen and virological response

Studies have shown that non-responders to HCV treatment have a greater incidence of DM and IR (28, 58). In a study of 234 patients with chronic HCV infection who were treated with IFNα ± ribavirin (RBV) and were followed for at least 3 years, impaired fasting glucose was found in 34.1% of the non-sustained responders versus 14.6% of the patients with sustained virological response (SVR) (28). SVR is defined as undetectable viral load after 24 weeks of treatment. Furthermore, none of the patients with SVR developed DM while nine of the non-responders eventually developed T2D. In another study, treatment failure for HCV was associated with an OR of 2.81 for the development of new-onset IR (59) while other groups have demonstrated improvements in IR after viral clearance, strongly suggesting causality (i.e., that HCV is responsible for the IR) (28, 60). It is unclear, however, if IR is simply a consequence of poor response to IFN–RBV therapy or if it, on the contrary, mechanistically contributes to treatment resistance. Conversely, there is very strong evidence that hepatic disease progression and response to antiviral therapy are worse with concomitant DM (28, 54, 61) (see Virological Response to Antiviral Therapy). This two-way association between hepatitis C and diabetes further supports a potential direct role of HCV in IR and T2D.

#### Mechanisms

Hepatic fibrosis and cirrhosis are not the only mechanisms leading to glucose intolerance and frank DM in HCV patients (27, 62). It may be assumed that HCV infection could primarily predispose to T2D through the induction of hepatic steatosis. Chronic HCV infection after all carries a higher prevalence of steatosis (50%) compared to other liver diseases, including chronic HBV infection (18%) and autoimmune liver disease (16–21%) (63). Viral steatosis, however, is most frequently present in chronic infections with HCV genotype 3 and yet genotype 3 is the least associated with IR. Studies also suggest that viral steatosis may behave differently from metabolic steatosis and that the steatosis seen in non-genotype 3 HCV infections is more a marker and consequence of the underlying IR (27). There is growing evidence demonstrating that specific and direct effects of the HCV itself are responsible for triggering the development of T2D.

##### Increase in reactive oxygen species

The HCV genome comprises 10 mature proteins, including structural proteins (core, E1, E2, p7) and non-structural proteins (NS2–NS5). Various *in vitro* studies have demonstrated chronic background inflammation and an increase in mitochondrial reactive oxygen species (ROS) in HCV infection. NS3 and NS5, in particular, were shown to trigger oxidative stress responses. Human monocytes incubated with various HCV proteins demonstrated that NS3 selectively generated ROS by activation of NADPH oxidase, Nox2 (64). Human hepatoma Huh-7 cells transfected with an NS5A vector have shown an elevation of ROS with consequent activation of STAT-3 and NF-KB pathways (65) that then led to the release of an array of cytokines, including TNFα, TGFβ, IL-6, and IL-8. The structural core protein has also been demonstrated to induce an increase in ROS, mitochondrial dysfunction, and ER stress by possibly overwhelming glutathione stores and ER chaperones during viral replication (66, 67).

##### TNFα and other inflammatory cytokines

The intense inflammatory response to HCV is deemed central to the development of peripheral and hepatic IR in chronic HCV infection, primarily through disruptions in the insulin signaling pathway. Several studies have reported that TNFα can directly interfere with insulin signaling in HCV patients (68–70). Knobler and colleagues noted significantly more detectable serum TNFα (measured as soluble TNFR1 and 2) in diabetic HCV+ patients than in non-diabetic HCV+ patients (respectively, 74 versus 64%; *p*-value <0.0001), independent of prior IFNα treatment or cirrhosis (71). CRP was higher in both diabetic groups (regardless of HCV seropositivity) while levels of IL-6 and IL-8 remained consistently low. Similar upregulation of TNFα and downregulation of IL-6 were observed in hepatic tissues of chronically infected HCV patients (72). Numerous animal and human studies with T2D patients have positively correlated TNFα with obesity and IR (73–76), and have implicated a role for TNFα in the pathogenesis of IR primarily through its postreceptor effects (71, 77). Animal pancreatic clamp studies have linked TNFα to increased IR and decreased glucose uptake (75). Similarly, *in vitro* studies using cultured adipocytes stimulated with insulin showed that chronic TNFα exposure reduced tyrosine kinase activities and decreased autophosphorylation of the insulin receptor and tyrosine-phosphorylation of insulin receptor substrate 1 (IRS-1) (71, 77, 78). Moreover, incubation of Huh-7 hepatocytes with TNFα increased serine-phosphorylation of IRS-1 (79), also resulting in inhibition of the insulin signaling cascade. TNFα also downregulated GLUT4 mRNA expression in muscle and adipose tissues and has been implicated in reduced expression of IRS-1 and PPARs (80, 81). A few studies have questioned the role of TNFα in IR and have shown no significant change in insulin signaling in skeletal muscle exposed to TNFα (82).

##### Direct alterations in insulin signaling by HCV

**Figure 1:**
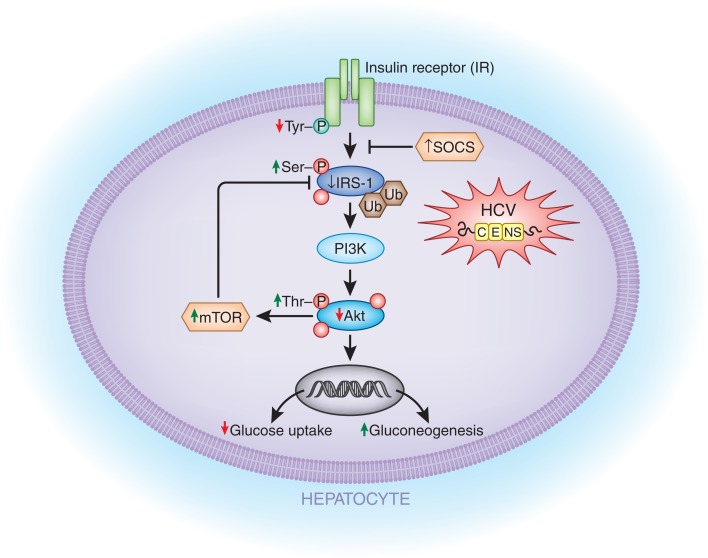
**Potential mechanisms by which HCV directly affects the insulin signaling cascade**. HCV infection of liver cells can lead to (1) decreased insulin receptor (IR in the figure) auto phosphorylation; (2) decreased IRS-1 activation due to increased serine-phosphorylation of IRS-1; (3) decreased IRS-1 levels due to increased ubiquitin-mediated proteasomal degradation induced by SOCS 3/7 and mTOR upregulation; (4) reduced Akt activity due to increased threonine-phosphorylation of Akt; (5) decreased GLUT4 expression; and (6) increased gluconeogenic enzymes (GC6P and PCK2). IR, insulin receptor; IRS-1, insulin receptor substrate-1; SOCS 3/7, suppressor of cytokine signaling; PI3K, phosphoinositide 3-kinase; mTOR, mammalian target of rapamycin; GC6P, glucose-6-phosphatase; PCK2, phosphoenolpyruvate carboxykinase 2.

There is increasing evidence that HCV has direct effects on insulin signaling (Figure 1). One study found that, compared to control livers, the livers of the non-obese HCV patients showed a twofold decrease in insulin-stimulated tyrosine-phosphorylation of IRS-1 and a significantly blunted activation of two downstream targets that are critical for most of the metabolic effects of insulin: phosphoinositide 3-kinase (PI3-kinase) and Akt (Protein Kinase B, a downstream target of PI3-kinase) (37). An imbalance in the levels of activating tyrosine-phosphorylated IRS-1, the inhibitory serine-phosphorylated IRS-1, and threonine-phosphorylated Akt seems to play an integral part in the development of IR in hepatitis C (79, 83, 84).

Another group demonstrated that mice transgenic for the HCV core protein showed decreased expression of IRS-1 and IRS-2 (85). This group further showed that there was an accumulation of ubiquitin-conjugated IRS-1 and IRS-2 in HepG2 human hepatic cells transfected with the HCV core protein. Proteasome-mediated degradation was further suggested by the increased IRS-1 and IRS-2 expression levels after treatment with MG 132, a proteasome inhibitor (84, 85). The inhibition of IRS-1 may be due to mTOR activation by the HCV 2a core protein which then ultimately leads to IRS-1 degradation (84).

These findings were replicated in another *in vitro* study using Huh-7 cells transfected with the HCV 1b or 3a core proteins (86). Ubiquitin-mediated proteasomal degradation of IRS-1 via SOCS-7, a negative regulator of IRS expression, was found only in the cells transfected with the HCV 3a core protein. These HCV 3a transfected cells also had significantly reduced mRNA levels of PPARγ, an insulin-sensitizing nuclear receptor, and showed an inhibition in insulin-induced Akt phosphorylation. When subsequently treated with a PPARγ agonist, not only were these effects reversed but also IRS-1 levels were noted to be increased (86). These results suggest that there may be genotype-specific mechanisms, although other groups have shown HCV 1b-mediated downregulation of IRS-1 by SOCS1 and SOCS3 (85).

##### Beta cell dysfunction

Histological evaluation of the pancreases of HCV-seropositive patients has shown evidence for pancreatic β-cells that are infected with HCV. These HCV-infected beta cells have been noted to have both morphological and functional defects, including a blunted insulin response to glucose (62). Narita’s group examined 131 chronic HCV patients with normal fasting serum glucose levels who were later diagnosed as either glucose tolerant, glucose intolerant, or overt diabetic after a 75 g oral glucose tolerance test (OGTT). These patients were further evaluated for various parameters of IR and of beta cell function (HOMA-β, C-peptide and insulin levels, ΔC-peptide 30, insulinogenic index). Significant decreases in the ΔC-peptide 30 and the insulinogenic index, both markers of early phase pancreatic insulin secretion, were consistently noted in the diabetic group suggesting a direct effect of HCV on beta cells (87). Others have suggested that proinflammatory cytokines secreted in chronically infected HCV patients, such as TNFα, may also affect beta cell function by disrupting insulin signaling/secretion and by sensitizing the beta cell to the toxic effects of free radicals (76, 88). These findings support the notion that HCV infection induces significant beta cell dysfunction either directly or through cytokine release.

### IFNα therapy – insulin resistance and type 2 diabetes

The literature is mixed on the effects of IFNα on IR. Some studies have shown an improvement in glucose levels during treatment of HCV infection. Huang et al. observed that 63 out of 180 hepatitis C patients with pre-diabetes (34.8%) became normoglycemic after PegIFNα–RBV therapy, while 10 (5.5%) developed DM (89).

It has also been documented that SVR may be an important factor in the reduction of IR after IFNα therapy (89, 90). The results from a cohort that included patients without diabetes, who were treated with IFNα for hepatitis C, showed that 143 out of 2,842 patients developed T2D during a mean observation time of 6.4 years. Predictive factors for development of diabetes in this study were older age, histological changes (cirrhosis), and non-SVR (60).

Also, injection of IFNα to healthy persons impaired glucose tolerance and insulin sensitivity, stimulated counter regulatory hormone secretion, and stimulated insulin clearance (91). In contrary, Ito et al. could not reveal any effect on insulin sensitivity and glucose tolerance at 3-month follow-up after IFNα treatment for hepatitis C (92).

Oral administration of low-dose IFNα (5,000 U) to patients with newly diagnosed T1D was shown to maintain more beta cell function after 1 year compared to high-dose (30,000 U) groups (93, 94). However, these studies had methodological limitations and there were no statistically significant differences in HbA1c or insulin dose between the two groups (93, 94).

## Pancreatic Islets Autoimmunity – Type 1 Diabetes and Hepatitis C Virus Infection of the Islets

Even though HCV is a liver-tropic pathogen, the antigen and viral sequences have been detected in other organs, such as the thyroid (95) and the pancreas (62, 96). Using RT-PCR on pancreatic tissue removed at autopsy from HCV-infected individuals, Yan et al. showed the presence of HCV RNA in all nine pancreata tested (96). However, the viral load was lower than in the liver. These results were confirmed in 5/9 pancreata by *in situ* hybridization (ISH), and by immunohistochemistry (IHC) in 6/9 pancreata (96). The authors suggested that the infected pancreas might act as a reservoir for HCV and may play a role in persistence of infection. Conversely, it is plausible that the infection may cause pancreatic islet dysfunction and/or trigger islet autoimmunity. Interestingly, epidemiological studies have shown a possible relationship between hepatitis C and pancreatic carcinoma (97), also suggesting direct infection of the pancreas by HCV and induction of local inflammation (98). If indeed direct infection of islets or acinar cells by HCV is confirmed, it is possible that it can trigger autoimmunity in susceptible individuals by inducing local inflammation with cytokine and chemokine secretion (also called “bystander mechanism”).

### HCV infection and type 1 diabetes

**Figure 2:**
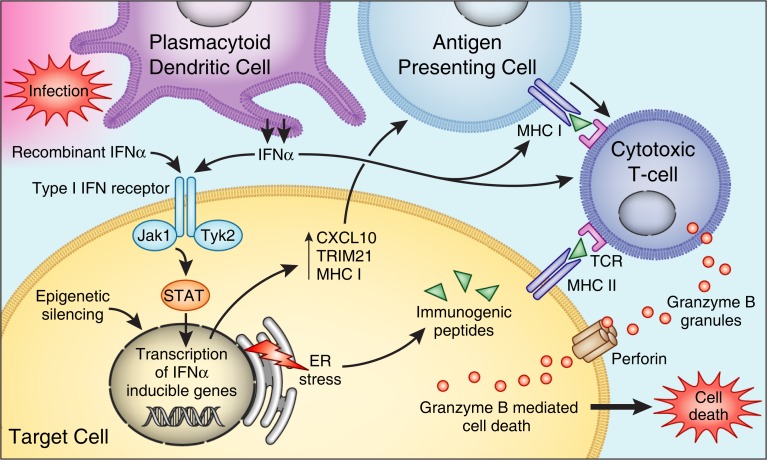
**Mechanisms by which IFNα affects beta cells to trigger islet autoimmunity**. PDCs produce IFNα in response to infection. Binding to IFN type I receptor, IFNα or therapeutic IFNα, activates Jak/STAT pathways – inducing transcription of IFN-inducible genes leading to (1) production of proinflammatory cytokines, (2) increase expression of HLA class I, (3) stimulation of cytotoxic T cells. IFNα also induces ER stress, and epigenetic modification of genes involved in the pathogenesis of type 1 diabetes.

Epidemiological studies have suggested but not confirmed an association between HCV infection and islet autoimmunity. In several studies, the frequency of T1D autoantibodies was not significantly higher in chronic HCV-infected patients selected for IFNα therapy than that in controls (99, 100). Hieronimus et al. reported the presence of glutamic-acid decarboxylase antibodies (GAD-antibodies) in 1 out of 47 untreated HCV patients with known T1D (99). Piquer and colleagues showed the presence of GAD-antibodies in 4 out of 277 (1.4%) non-diabetic HCV patients, and 1 out of 273 (0.4%) in control population. However, this difference did not reach statistical significance (101). Nevertheless, there are several cases of T1D reported in the literature that were associated with acute hepatitis C infection. Chen reported a case of a male patient who developed acute hepatitis C after blood transfusion. The patient presented GAD-antibodies and islet cell antibodies (ICA Abs) 4 weeks after transfusion, and developed T1D 1 year later (12). The patient had HLA DR3 and DR7, which are not associated with T1D in China. Another case, a 22-year-old female with type 1 diabetes and acute hepatitis C, was reported by Masuda (13). Therefore, it seems that the majority of the risk for T1D in hepatitis C patients is due to IFNα therapy (see below); however, HCV infection itself may also contribute synergistically to the association, possible by local production of IFNα in the islets if HCV indeed is proven to infect the islets (Figure 2).

### IFNα therapy in HCV infection and type 1 diabetes

Interferon alpha was discovered more than 50 years ago (102), and is now recognized as a key component of the innate immune response and the first line of defense against viral infections. IFNα therapy was shown to be particularly effective in HCV infection. Indeed, hepatitis C is the most frequent indication for IFNα therapy (103), even though recently non-interferon-based therapies for HCV infection have been replaced interferon-based therapies. IFNα treatment has considerably improved the outcomes in patients with chronic HCV infection, however, with its potent immunomodulatory effect it may contribute to development of autoimmunity.

#### Epidemiology

The first case of IFNα-induced T1D was reported by Fabris et al. (104). Although the exact incidence of IFNα-induced diabetes is unknown, several large retrospective studies investigating adverse effects of IFNα in patients with chronic viral hepatitis, mainly hepatitis C, reported incidences of 0.08% (10/11.241) (105) in Italy, and 0.7 (5/667)–0.96% (12/1.250) in Japan (106, 107). Nakamura et al., in a nationwide study in Japan, identified 91 patients with IFNα-induced T1D (108). Furthermore, a Dutch study reported development of T1D in 5 out of 189 patients (2.6%) treated with Peg-IFN + RBV (109). In a study from Spain, including 46 patients, no evidence of increased prevalence of beta cell autoimmunity was found 6 and 12 months after IFNα therapy (101).

In most reported cases, the onset of T1D occurred during or shortly after treatment with IFNα [reviewed in Ref. (110)]. To date, there are more than 45 case reports of T1D developing after treatment with IFNα. The clinical presentation is usually of an acute onset as is typical for T1D (108). The levels of GAD-antibodies were reported to be significantly higher in IFN-induced T1D compared to classical T1D (106). It is important to suspect T1D in HCV patients experiencing polyuria and polydypsia, or other T1D symptoms after initiation of IFNα therapy. Of note, treatment with IFNα is associated with many side effects such as flu-like symptoms, gastrointestinal (GI) symptoms, and headaches (111) that can mask the symptoms of diabetes, resulting in delayed diagnosis and complications.

#### Risk Factors for IFN-Induced Type 1 Diabetes

##### Age and gender

Whether age is a risk factor for IFN-induced T1D is difficult to answer since the age of onset of T1D in HCV patients likely reflects the age at which IFNα was initiated. The mean age at the onset of IFN-induced T1D was 56 years in a nationwide survey in Japan that included 91 patients who developed interferon-induced T1D (most received IFNα for HCV infection) (106).

The reported female to male (F/M) ratio of IFN-induced T1D in the same survey in Japan was 0.90, which is lower than the 1.4–1.5 F/M ratio for T1D in the Japanese population (106) and likely reflects the male preponderance of HCV. Another study also found no significant difference in the male and female frequency in children or adults who developed IFN-induced T1D (112). Similarly, the F/M ratio for IFN-induced T1D in Europe (~0.5) was also lower than that for classical T1D (~1) (105, 109). This ratio may reflect the gender distribution of hepatitis C as numerous large cohorts reported that overall two-third of reported cases of chronic HCV infection were males [for review, see Ref. (113)].

##### Preexisting islet autoantibodies

Autoantibodies are often reported in both adults and children with HCV infection. Wesche et al. reported development of islet autoantibodies in 9.7% (6/62) of patients after initiation of IFNα therapy (114). In another cohort, 2.5% (5/186) non-diabetic patients who received Peg-IFN + RBV developed T1D, and 1.6% (2/186) developed T2D (109). Two out of the five patients with IFN-induced T1D were tested positive for islet antibodies at the baseline prior to initiation of interferon therapy. Results from a recent cohort reported development of T1D in 1 out of 114 children (age 4–17 years) with chronic hepatitis C treated with Peg-IFN, while 9.3% had positive diabetes-related antibodies at baseline (4% with GAD-antibodies) (112).

Even though islets autoantibodies, diagnostic of T1D, have not been commonly measured pre- and post-treatment with IFNα, seroconversion has been observed following IFNα therapy. Fabris reported clinical features of 31 cases of IFN-induced T1D of whom 23 were treated for chronic hepatitis C (1992–2002) (16). For those who were tested for antibodies, markers for pancreatic islet autoimmunity were present prior to IFNα therapy in 50% of cases with IFN-induced T1D. These data are consistent with the well-known fact that islet autoantibodies are the best biomarkers of genetic risk factors for T1D. These findings are similar to those observed for IFNα-induced thyroiditis where thyroid Abs were shown to be the strongest risk factor for IFN-induced thyroiditis (115).

##### Family history

One study that examined family history in patients that developed T1D during or following IFNα therapy did not find history of T1D in first degree relatives of patients with IFNα-induced T1D (108). It is, however, interesting that 15% of patients with chronic HCV infection had T2D, and 23% had first degree family history of T2D (108). Among 31 cases of IFN-induced T1D reported by Fabris, family history of T1D was reported in 3/31 (9%) patients who developed IFN-induced T1D and family history of T2D was reported in 6/31 (19%) (16). Koivisto postulated that the impairment in insulin sensitivity (91), and the resulting stimulation of beta cells may make islets more vulnerable to destructive process of IFNα therapy (116).

##### Viral genotype

The HCV genome was determined by Choo et al. (117). Based on the identification of genomic differences, HCV has been classified into distinct subtypes. Genotype 1 (1a and 1b) is the most common form in the USA and Europe (118), while genotype 1b is common in Japan. Genotype 2 is the second commonest strain in North America, Europe, and Japan (119, 120). Genetic heterogeneity of HCV may account for some of the differences in disease outcome and response to treatment observed in HCV-infected individuals (121).

In the Japanese nationwide survey, genotype 1b was most frequently detected (81%) in patients that developed IFN-induced T1D (106), but this likely reflected the fact that genotype 1b is the most prevalent HCV subtype in the Japanese (121) and Chinese populations (122). Patients with genotype 1 that is more resistant to therapy, usually receive higher doses of IFNα and for longer duration contributing to adverse effects (113). In a recent study, Nakanishi and co-workers could not find an association between genotype 1b and the development of T1D; however, the study was limited as the control group included very few patients (106). Similarly, Hasham et al. reported that 74% of patients with IFN-induced thyroiditis were infected with HCV genotype 1, however, 76% of hepatitis C controls also had genotype 1 (123). Thus, viral genotype does not contribute to the association of interferon therapy in HCV patients with T1D.

##### Therapeutic regimen and virological response to therapy

Data regarding the impact of therapeutic regimen and SVR on development of IFN-induced diabetes are difficult to compare as different forms, dosages, and durations of IFN therapy were used in different studies. Until 2011, the standard IFNα regimen for HCV infection included pegylated IFNα (peg-IFN) 2a in combination with RBV, with genotype-dependent dosage and duration (124). Genotype 1 has the lowest SVR to this regimen compared to genotypes 2 and 3, and it requires higher doses of IFNα. This might contribute to more side effects (113, 125). RBV also has immunomodulatory effects, but RBV is rarely used as monotherapy. However, there is a case report describing a patient who developed T1D after RBV was added to Peg-IFN (126).

The median time from initiation of IFN therapy to the onset of T1D was 8 months in a Japanese cohort, and was significantly shorter in those who were treated with the Peg-IFNα in combination with RBV compared to those who were treated with non-Peg-IFN in mono therapy (108). Interestingly, almost 60% of patients who developed T1D were negative for HCV RNA at the onset of T1D. Overall, the mean latency period in reported cases of IFN-induced T1D was 5 months and ranged from 10 days to 4 years [reviewed in Ref. (16)]. However, no correlation was found between the rate of virological response and the latency of development of T1D (16, 108). Supporting this observation is an Italian cohort that did not show a significant difference in the incidence of glucose abnormalities between long-term responders and non-responders (58).

The current guidelines for treatment of chronic HCV infection recommend using protease inhibitors in combination with Peg-IFN with or without RBV for genotype 1 (127). However, protease inhibitors have side effects too, and recently a case of T1D induced shortly after triple therapy was reported (128). Further studies are needed to determine the impact of protease inhibitors in combination with IFN, or in monotherapy, on the development of autoimmunity. In addition, as the therapy for chronic HCV infection switches to non-interferon-based therapies, it is possible that T1D will no longer be triggered by therapy for hepatitis C.

##### Genetic factors

Genetic susceptibility plays a major role in the etiology of T1D (129). Therefore, genetic predisposition may also play a role in the etiology of IFNα-induced T1D. In a Japanese cohort, HLA-A*2402 [associated with T1D in Japan (130)] was shown to be associated with the development of T1D in patients treated with IFNα (106). It is intriguing that HLA-A24 has also been shown to be associated with the development of IFNα-induced thyroiditis in Japan (131), suggesting a general role for HLA-A24 in IFNα-induced autoimmunity. The frequency of HLA-DR4 and DR9 (also susceptibility genes for T1D in Japan), as well as DR13, was also significantly higher in patients with IFNα-induced T1D when compared to healthy controls (108). Moreover, HLA-DR13 was also significantly more frequent in patients with IFNα-induced T1D compared to patients with classical T1D, suggesting a direct role in IFNα-induced autoimmunity through an environmental–genetic interaction (108). Studies and results from reported cases, including patients with different ethnicity, showed association between IFN-induced T1D and classical T1D HLA haplotypes (DR3, DR4, DQ8) (16, 109, 110).

Interferon alpha may also trigger or accelerate disease development in genetically predisposed individuals. We previously showed that injecting IFNα to NOD H2h4 mice, a strain genetically susceptible to spontaneous autoimmune thyroiditis, caused a higher frequency of autoimmune thyroiditis. These findings support the notion that IFNα triggers or accelerates autoimmunity in genetically susceptible individuals (132). However, similar studies have not been performed in mice genetically predisposed to autoimmune diabetes.

Taken together, these data support two potential mechanisms by which genetic susceptibility can contribute to IFN-induced T1D: (1) some individuals inherit classical T1D susceptibility genes and IFNα simply triggers or accelerates the progression of T1D in them; alternatively, (2) other individuals inherit genetic variants that uniquely predispose to IFNα-induced autoimmunity.

### Mechanisms

The mechanisms by which IFNα can induce T1D have not been thoroughly studied (Figure 2). IFNα has diverse effects on the immune system that can be implicated in the development of autoimmunity. By binding to its receptor, IFNα activates the JAK–STAT pathway (133–135), leading to expression of interferon-stimulated genes (ISGs), including cytokines and adhesion molecules genes. These proinflammatory cytokines can trigger autoimmunity in genetically susceptible individuals (Figure 2).

A direct effect of IFNα on the pancreas might also trigger T1D. Indeed, several studies have shown elevated levels of IFNα in the pancreas of patients with a recent onset of T1D (136–138). Transgenic mice over-expressing IFNα in their beta cells developed hypoinsulinemic diabetes associated with islets inflammation, demonstrating that IFNα can trigger T1D locally by direct effects on the islets. This inflammatory disease process was prevented through the use of an IFNα neutralizing antibody (139). Our group has previously shown similar results in transgenic mice over-producing IFNα in the thyroid; these transgenic mice developed severe inflammatory thyroiditis (140). In our thyroidal model, IFNα triggered the recruitment of inflammatory cells through the induction of various cytokines and chemokines, and it activated cytotoxic T cells causing thyroid cell necrosis (Figure 2). Moreover, we were also able to show direct toxic effects of IFNα on thyroid cells (140). If a similar mechanism occurs in the islets, it is possible that IFNα can also induce toxic effects in islet cells. Li et al. demonstrated that blockade of IFNα signaling in 2- to 3-week-old NOD mice by anti-IFNAR1 mAb resulted in delayed onset and decreased incidence of diabetes (141). This was accompanied by an increase in the number of immature dendritic cells in draining popliteal lymph nodes and enhanced production of the cytokines IL-4 and IL-10. Furthermore, interferon regulatory factor 1-deficient NOD mice were protected from T1D (142).

Interferon alpha modulates the ability of immune effector cells to interact with infected cells, partly through upregulation of MHC class I molecules on target infected cells (143). Moreover, overexpression of MHC class I antigens is associated with activation of cytotoxic T cells. Upregulation of MHC class I molecule expression on islets cells is a prominent, early feature during the development of T1D (144), and it is possible that this is mediated by IFNα. In one study, 33 of 34 pancreata removed at necropsy from patients with T1D revealed beta cells that were positive for immunoreactive IFNα and showed hyperexpression of MHC class I antigens (136). Moreover, type I interferons decrease regulatory T cell activation, another effect that may play a role in development of autoimmunity (145, 146). In contrast to these data, a recent study showed an increase in the number of regulatory T cells and a decrease in CD8+ T cells in the livers of mice treated with IFNα (147).

## Effects of Insulin Resistance and Type 2 Diabetes on Outcome of Chronic HCV Infection

While HCV infection has been demonstrated to be a risk factor for T2D (see Type 2 Diabetes and Insulin Resistance in HCV Infection), patients with HCV and IR have also been shown to have significantly worse clinical outcomes. This two-way association further exemplifies the integral role of IR in HCV infection. IR is associated with increased liver fibrosis, reduced virological response to antiviral therapy, and higher rates of HCC. There is also some evidence that IR increases the risk for liver-related death and is associated with shorter time to transplantation (148).

### Association of diabetes/insulin resistance with worse outcomes in hepatitis C

#### Hepatic Fibrosis and Cirrhosis

A number of factors are known to increase the risk of liver fibrosis and subsequent cirrhosis. These include longer duration of infection, older age at time of exposure, male gender, co-infection with other viruses, and alcohol consumption (149, 150). In addition to these factors, IR is increasingly recognized as a separate risk factor for fibrosis progression and cirrhosis.

Two studies by Hui et al. demonstrated that IR is an independent predictor of fibrosis in chronic HCV infection (54, 151). Similarly, Hsu et al. found a strong association between diabetes and severity of fibrosis (152). A recent, large cohort study from Taiwan also concluded that patients with chronic HCV infection who developed diabetes had a two to threefold increased risk for development of cirrhosis and hepatic decompensation (57). However, these findings are not uniform and a few studies failed to show this correlation (153–155). While Svegliati-Baroni et al. did not find an association between HOMA-IR and liver fibrosis, they did find post-load IR to be a predictor of fibrosis progression correlation (153). Overall, the majority of studies support the notion that IR and diabetes predispose to liver fibrosis and cirrhosis.

#### Virological Response to Antiviral Therapy

While there is significant evidence to suggest that DM worsens response to antiviral therapy, a few studies could not confirm this relationship. A large study of 330 patients found that reduced HOMA-IR correlated with lower rates of SVR, particularly in patients with “difficult to treat” chronic HCV populations, including cirrhotics, the overweight, and HIV-coinfected individuals (156). Other studies suggested that HOMA-IR only predicts treatment response in patients with genotype 1 HCV (157). An Italian study of 412 patients showed a trend for association (albeit not statistically significant) between higher HOMA-IR and reduced SVR (*p* = 0.06) (158).

To resolve these conflicting data, a recent meta-analysis of 14 studies showed that patients with IR treated with pegIFN–RBV had a 20% lower rate of SVR compared to patients without IR (95% CI: −29.9 to −9.4%, *p* < 0.001). In addition, responders had a lower HOMA-IR compared to non-responders (mean difference: −0.92, 95% CI: −1.53 to −0.32, *p* < 0.001) (159).

#### Hepatocellular Carcinoma

Hepatocellular carcinoma is the third leading cause of cancer-related mortality worldwide (160). Chronic HCV infection is an established risk factor for HCC. Recent studies suggest that diabetes also increases the risk of malignancy, including HCC (148, 161, 162). Additionally, diabetes is a risk factor for HCC-related mortality (163).

A recent Taiwanese study found that HCV patients with diabetes had a hazard ratio of 1.73 for HCC compared to non-diabetic HCV patients (161). Of note, patients with hepatitis B did not demonstrate a similar increase in HCC incidence (161). Another study of HCV patients with Ishak fibrosis score 4–6 found HCC incidence to be 11.4% in diabetics, and 5.0% in non-diabetics (162). The relationship persisted even when controlling for albumin, bilirubin, and platelet count. The degree of IR also appears to correlate with HCC risk. A study from France in patients with HCV cirrhosis found the 5-year incidence of HCC to be 7, 18, and 27% for the lowest, middle, and highest tertile of HOMA-IR, respectively. In a multivariate analysis, HOMA-IR was still predictive of HCC incidence, albeit with a hazard ratio of 1.1 (148).

While the majority of published studies support an association between diabetes and HCC in HCV patients, there are a few reports showing otherwise. However, a 2006 literature review found an association between diabetes and HCV-related HCC in 9 of 13 case-control studies and 7 of 13 cohort studies (164). Despite some inconsistencies, the pooled risk ratio and pooled OR were both significant at 2.5.

### Mechanisms of accelerated disease progression in HCV patients with diabetes and insulin resistance

#### Hepatic Fibrosis and Cirrhosis

Liver fibrosis is a reversible wound-healing response to hepatic cell injury. Parenchymal cell regeneration in response to acute injury generally results in minimal deposition of extracellular matrix (ECM). However, chronic hepatic injury disrupts the balance between liver repair and scar formation. Hepatocytes are replaced with excessive ECM produced primarily by hepatic stellate cells (HSC) (165).

Excessive HSC activation is one proposed mechanism by which IR can induce fibrosis. Supporting this hypothesis, *in vitro* studies showed that hyperglycemia and hyperinsulinemia upregulate the secretion of connective tissue growth factor mRNA, a growth factor involved in HSC activation and subsequent fibrogenesis (166). In addition, insulin can directly stimulate HSC proliferation in a dose-dependent fashion (167).

Hepatitis C virus is associated with accelerated steatosis that is mediated through increased production of lipogenic substrate, upregulation of lipogenesis, and disruptions of fatty acid metabolism (168). The magnitude of HCV-related steatosis is compounded by IR. IR activates the lipid biosynthetic pathway in the liver resulting in dyslipidemia and increased steatosis, which can accelerate liver fibrosis (169). Decreased insulin sensitivity also increases release of free fatty acids from adipose leading to increased hepatic lipid deposition (170). Enhanced hepatic lipid deposition and steatosis are independently associated with increased fibrosis in patients with genotype 1 HCV (171).

Lastly, a reciprocal regulation between HCV and SNF1/AMP kinase-related kinase (SNARK) promotes TGF-β signaling, a major driver of hepatic fibrogenesis. *In vitro* studies have shown that metformin suppressed both HCV replication and SNARK-mediated enhancement of TGF-β signaling (172).

#### Virological Response to Antiviral Therapy

IR is associated with increased levels of protein tyrosine phosphatases (PTP). PTPs inhibit phosphorylation of signal transducers and activators of transcription (STATs), thus preventing activation of a group of key transcription factors in the interferon signaling pathway that can lead to decreased effectiveness of IFNα therapy and lower rates of SVR (173, 174). This has been corroborated by an *in vitro* study that demonstrated an insulin-driven dose-dependent decreased expression of IFN-stimulated genes, including PKR, MxA, and OAS-1 (175).

*In vitro* studies have shown that metformin downregulates PTP-1B and improves response to IFNα in liver cell lines (174), and that metformin inhibited HCV replication by activating AKT (176). However, clinical trials examining the role of metformin in antiviral treatment regimens provided mixed results (157). The TRIC-1 study group compared metformin versus placebo in HCV patients treated with pegINF–RBV. While intention to treat analysis showed no effect of metformin, per protocol analysis displayed a 10% absolute increase in treatment success with metformin (157). Moreover, subgroup analysis of females showed an SVR of 57.7% with metformin versus 28.6% with placebo (*p* = 0.03). A recent open-label study by Yu et al. found that addition of metformin to pegIFN–RBV resulted in 59.2% SVR compared to 38.8% SVR without metformin (177). In contrast, a placebo-controlled trial by Sharifi and colleagues found no correlation (178).

Additionally, HCV replication is thought to be downregulated by p21-activated kinase 1 (PAK1). IR may reduce PAK1 activity leading to upregulation of HCV replication (179, 180). IR is also associated with increased viral load (181) and progression of fibrosis (54, 152), which independently influence response to interferon-based therapy (182). It remains to be determined the extent to which SVR will continue to be affected by IR when using novel anti-HCV regimens.

#### Hepatocellular Carcinoma

Patients with diabetes are at increased risk for various types of malignancies (183–185). HCC appears to be one of the malignancies most strongly linked to diabetes with a relative risk of 2.5 (184, 185). Chronic HCV infection thus promotes HCC both directly and indirectly through the virus’s diabetogenic effects (184). While the exact mechanisms have yet to be elucidated, insulin, insulin-like growth factor (IGF), and chronic inflammation have all been implicated in tumorigenesis (184, 186). Additionally, hyperglycemia is thought to promote tumorigenesis by providing the metabolic substrate necessary for cancer cell survival (184).

**Figure 3:**
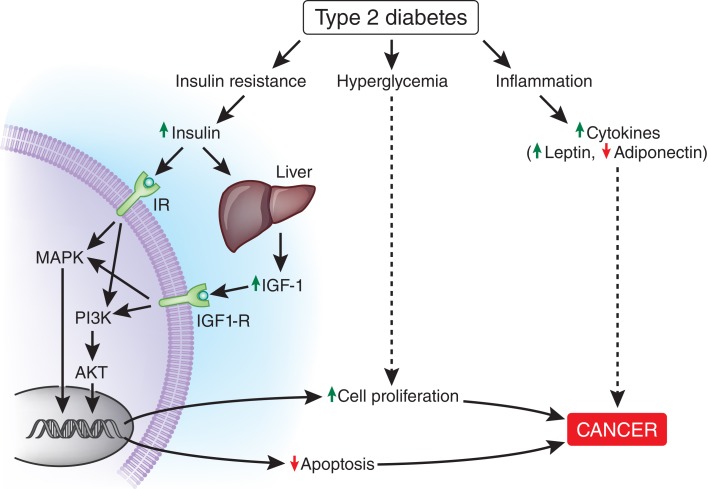
**Possible mechanisms linking diabetes to accelerated cancer development**. Binding of insulin to the insulin receptor (IR in the figure), likely IR-A and/or binding of IGF-1 to the IGF-1 receptor activate MAPK and PI3K/AKT signaling pathways leading to increased cell proliferation and decreased apoptosis. Hyperglycemia and chronic inflammation also contribute to cancer progression through other mechanisms. The mechanisms by which HCV promotes the development of HCC were not included in this figure. IGF-1, insulin-like growth factor 1; IGF-1R, insulin-like growth factor 1 receptor; IR-A, insulin receptor A; MAPK, mitogen-activated protein kinase; PI3K, phosphoinositide 3-kinase; AKT, protein kinase B.

The insulin receptor is regulated by alternative splicing mechanisms to yield two isoforms, IR-A and IR-B. IR-A expression is increased in cancer cells, including HCC, and primarily promotes the mitogenic role of insulin, while IR-B primarily controls insulin’s metabolic effects (187, 188). High insulin levels in the setting of IR likely activate the IR-A pathway driving tumorigenesis, while the IR-B pathway remains downregulated. This phenomenon would be amplified in the setting of IR-A upregulation, which occurs in cancer cells including HCC (187). Chettouh et al. demonstrated that HCC cell lines with enhanced IR-A expression proliferated in response to insulin while normal human hepatocytes did not (187). Insulin binds to its receptor, likely IR-A, in the setting of tumorigenesis, and activates downstream signaling cascades via insulin receptor substrate (IRS) proteins (189). Indeed, levels of IRS proteins are elevated in human cancers, including HCC (181). IRS proteins can activate mitogen-activated protein kinase (MAPK) and the phosphoinositol-3-kinase (PI3K)/AKT pathway (186, 188, 189). These pathways are involved in cell growth and proliferation and inhibition of apoptosis as shown in Figure 3 (186, 188, 189).

Hyperinsulinemia also augments IGF-1 receptor (IGF-1R) signaling through increased hepatic production of IGF-1 and decreased hepatic production of IGF-binding proteins (IGF-BPs), thereby increasing levels of free IGF-1 (184, 190). Similar to activation of IR-A, IGF-1R activation leads to stimulation of the MAPK and PI3K/AKT pathways (Figure 3) (184, 186, 189). The LeRoith group demonstrated that administration of IGF-1 to liver-specific IGF-1-deficient (LID) mice with orthotopically implanted colon adenocarcinoma resulted in increased liver metastasis compared with saline controls (191). Therefore, it is possible that elevated levels of IGF-1 induced by hyperinsulinemia could also promote HCC growth. Notably, IGF-1 and IGF-BPs are primarily produced in the liver. Greater than 90% of patients with HCC suffer from cirrhosis (192), which may impair IGF-1 and IGF-BP synthesis.

Diabetes and obesity are states of chronic inflammation associated with elevated levels of leptin and low levels of adiponectin (193). Leptin is a proinflammatory adipokine that is linked to cell proliferation and invasion of cancer cells, while adiponectin is an anti-inflammatory adipokine that has been linked to reduced tumor growth (184, 188, 193). While the precise relationship between leptin, adiponectin, and HCC is still poorly understood (194), the chronic inflammation associated with diabetes may also promote HCC.

Further supporting the association between HCC and diabetes is recent evidence that metformin decreases the risk of HCC in patients with chronic HCV infection. A 2009 study of patients with HCV cirrhosis and diabetes found the 5-year incidence of HCC to be 9.5% for patients treated with metformin versus 31.2% without metformin therapy (*p* = 0.001) (182). Several additional studies, including a recent meta-analysis, show similar findings (183, 184). While clinical data are suggestive that metformin has anti-neoplastic properties, supporting laboratory models have been criticized for using concentrations of metformin that greatly exceed clinical dosing (195). Further studies are needed to determine whether metformin’s anti-neoplastic effects are secondary to direct effects of metformin on hepatocytes or due to the insulin-sensitizing effect of metformin.

## Screening for Diabetes in HCV Patients and Therapeutic Considerations

### Screening

#### Who?

As summarized above, epidemiological data clearly show that patients with chronic HCV infection are more susceptible to develop T2D. Moreover, there is negative impact of IR and diabetes on virological response and long-term outcomes of HCV infection. Therefore, we recommend screening for diabetes in all HCV-infected patients regardless of age or medical history. There is also epidemiological evidence for increased prevalence of hepatitis C in patients with diabetes, and some authors find it reasonable to screen patients with diabetes and persistently elevated ALT for HCV (196). T2D patients frequently present with abnormal liver enzymes (197); however, in most cases, the elevated ALT levels may represent non-alcoholic fatty liver disease (NAFLD). Still testing for hepatitis C could be considered in diabetic patients with elevated liver enzymes, especially in cases with no evidence of NAFLD. We recommend that each case be evaluated individually.

**Figure 4:**
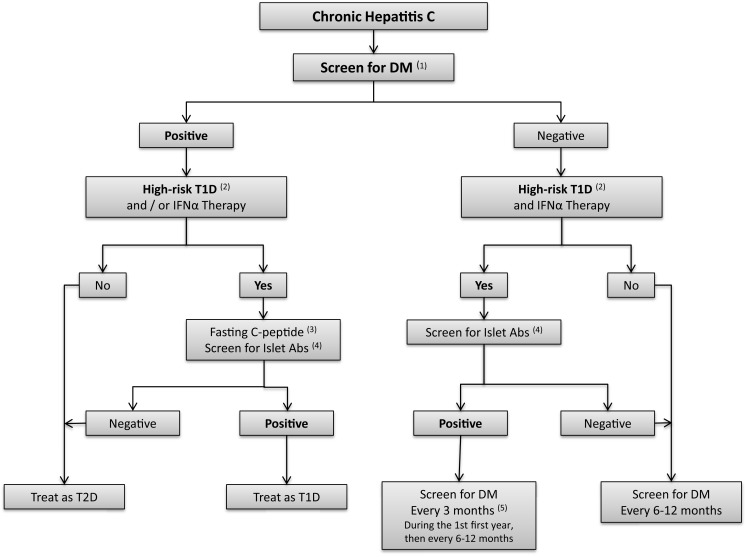
**Algorithm for screening for diabetes in hepatitis C patients**. (1) *Screen for DM*: fasting blood sugar (FBS) and/or oral glucose tolerance test (OGTT). HbA _1_c might be considered if the measurement methods are well established. (2) *High risk Type 1 diabetes patients* are defined as individuals with first degree relatives with type 1 diabetes (T1D); patients with other autoimmune conditions; or patients might be considered as high risk, if IFNα therapy is considered. (3) Fasting C-peptide levels. (4) *Islet antibodies include* autoantibodies to glutamic-acid decarboxylase (GAD65), autoantibodies to insulin (IAA2), autoantibodies to the tyrosine phosphatase IA-2 and IA2B, autoantibodies to zinc transporter 8 (ZnT8), (5) IFNα may induce autoimmunity and therefore measure­ment of islet antibodies should be considered before and after IFNα therapy in high risk patients, and in patients with suspicion of T1D after initiation of IFNα.

Whether screening for islet autoantibodies is necessary prior to IFN therapy, which has been shown to trigger islet autoimmunity, is unclear. Fabris reported the presence of autoantibodies in 50% of patients with IFN-induced T1D (16). In our opinion, screening for islet antibodies should be considered in patients with increased risk for T1D, such as patients with first degree relatives with T1D, patients with known autoimmune conditions, and HCV patients receiving IFNα (Figure 4). Pretreatment screening for diabetes (prior to IFNα therapy) and periodic monitoring of blood glucose (BG) should be performed during and after IFNα treatment.

#### How?

According to ADA guidelines, the standard screening test for diabetes is fasting plasma Glucose (FPG) (198). The 75-gr oral glucose test (OGT) should be considered in patients with pre-diabetes (fasting blood sugar (FBS) 100–125 mg/dL or 6–6.9 mmol/L) or high risk of diabetes.

HbA_1_c is an important tool for follow-up of diabetic patients; however, recent prospective studies have shown a continuous association between HbA_1_c at baseline and subsequent diabetes. Therefore, HbA_1_c levels in the range of 5.7–6.4 can also be considered as pre-diabetes (198). Treatment with IFNα and RBV may induce hemolytic anemia, thereby falsely decreasing HbA1c levels (199). Testing for fructosamine may be valuable in these cases as well as in cases with known hemoglobinopathies.

#### How Often?

Screening for T2D is recommended every 3 years in the general population of individuals older than 45 years (198). In our opinion, screening for diabetes should be performed every 6–12 months in patients with hepatitis C in view of the strong association between the two diseases. We recommend testing for T1D if the patient develops diabetes after initiation of IFNα therapy (Figure 4).

### Therapeutic considerations

Diabetes in HCV patients should be treated according to the ADA guidelines (198). Management of diabetes in patients with liver diseases is, however, complicated by the effects of drug metabolism by the liver, drug interactions, and potential liver toxicity of some medications used to treat diabetes [For review, see Ref. (196)].

#### IFNα-Induced Type 1 Diabetes

To date, no consensus exists regarding continuation or withdrawal of IFNα–RBV in IFN-induced T1D. However, reported cases show that IFNα–RBV are frequently stopped after diagnosis of IFN-induced T1D, presumably in the hope of reversing the diabetes. Permanent insulin treatment has been required in most cases of IFN-induced T1D (16); however, improvement has been observed in a few cases (110).

#### Insulin Resistance and Type 2 Diabetes

Metformin was not reported to cause hepatotoxicity and therefore should be considered as the drug of choice in HCV patients with IR or T2D. Although there is no clear evidence that treatment with metformin will increase the sustained viral response in HCV patients, there is increasing evidence that metformin is independently associated with reduced risk for HCC and liver-related death/transplantation (157, 177, 178, 200). The use of metformin, however, is not recommended in advanced hepatic disease because of increased risk for lactic acidosis (201).

Use of Metformin for other liver pathologies associated with IR, such as NAFLD, did not improve hepatic histopathology (202). By contrast, recent studies on another class of insulin sensitizers – thiazolidinediones (TZDs) – in NAFLD showed improvement in ALT and liver histology [for review, see Ref. (203)]. Sumie et al. assessed the effect of pioglitazone in patients with HCV-induced HCC and found no effect on recurrence free survival (204). Nevertheless, the treatment improved the IR (204). Yet, due to reported cases of acute cholestatic hepatitis (205, 206), TZD use is not recommended in advanced liver cirrhosis.

Glucosidase inhibitors act directly on the GI tract and in a recent placebo-controlled cross over study in patients with grade 1–2 hepatic encephalopathy, acarbose decreased both fasting and postprandial glucose (207). Acarbose increases the risk of hyperammonemia (208), and can induce mild transient elevation of ALT. Rare reversible cases of severe liver disease have been reported [for review, see Ref. (209)], and therefore, it should be used with caution.

Glucagon-like peptide-1 (GLP-1) is a gut-derived incretin hormone that stimulates insulin secretion. Dipeptidyl peptidase-4 (DPP-4) inhibitors increase incretin hormone levels, such as GLP-1. Itou et al. demonstrated that the serum GLP-1 levels were significantly decreased in HCV patients compared to control group and HBV group; and that DPP-4 expression was significantly increased in the ileum, liver, and serum in the HCV group. The authors concluded that altered expression of GLP-1 may be involved in the development of HCV-associated glucose intolerance (210). Recent studies on GLP-1 have shown a slowing of the progression of NAFLD by direct effects on lipid metabolism in hepatocytes, and on inflammation in the liver. DPP-4 inhibitors may also affect hepatic pathways of fat elimination [for review, see Ref. (211, 212)]. A case-control study assessed the efficacy and safety of DPP-4 inhibitors in HCV patients (16 patients with T2D, included 3 patients with hepatic cirrhosis) and reported a reduction in HbA1C without side effects (213). Further larger studies are needed to support their use in patients with advanced hepatic diseases.

There are no trials examining the safety of sulfonylureas (SUs) in patients with chronic liver diseases. The use of SU with short half-life is generally considered safe in patients with liver disease (196). However, close monitoring of BG is necessary since there might be increased risk of hypoglycemia. SUs are metabolized by the liver and excreted by the kidneys, and their use is contraindicated in advanced liver diseases.

Insulin has been considered as the drug of choice in patients with diabetes and decompensated liver disease due to short half-life to prevent hypoglycemia and hyperglycemia by frequent adjustment of the doses. However, a nested case-control study conducted in Japan found that use of insulin and second-generation SU were independent variables associated with incidence of HCC (OR = 2.96 95% confidence interval 1.29–6.82) (214). Other studies showed that use of metformin was associated with reduced risk of HCC compared with SUs and insulin (215, 216). A meta-analysis of observational studies reported that insulin and SU increased the risk for HCC, while metformin reduced the risk, and TDZs did not change it (217). Prospective studies are required to assess the link between exogenous insulin therapy and SU, and the risk for HCC.

## Conclusion

Hepatitis C and DM are chronic diseases with high global prevalence of epidemic proportions. The association between hepatitis C and DM has been substantiated by numerous studies in the past two decades and represents a two-way association. On the one hand, HCV infection triggers diabetes, mostly type 2 but occasionally (especially in patients treated with IFNα) type 1 diabetes; and, on the other hand, diabetes worsens hepatitis C outcomes, including increasing the risk for cirrhosis and HCC. Mechanistic studies support the hypothesis that HCV infection leads to defects in insulin signaling pathways, and that direct effects of HCV on the beta cells are possible. Metformin is the drug of choice in chronic HCV infection as data provided show a significant reduction in the risk for HCC. Further studies are needed to assess the long-term effect of TDZs and DPP-4 inhibitors on glycemic control in HCV patients, and the effects of insulin and SU on cancer progression.

In the light of these data, it is essential that physicians caring for HCV patients are aware of the high risk for T2D and T1D and that they screen HCV patients for diabetes. In addition, the presence of diabetes in an HCV patient should alert the clinician to the possibility of worse outcomes. It remains to be determined whether the new non-interferon-based therapeutic regimens for chronic HCV infection (such as protease inhibitors) will reduce the frequency of diabetes in HCV patients or improve glycemic control if diabetes has already developed.

## Conflict of Interest Statement

The authors declare that the research was conducted in the absence of any commercial or financial relationships that could be construed as a potential conflict of interest.
